# Expression of AFP and Rev-Erb A/Rev-Erb B and N-CoR in fetal rat liver, liver injury and liver regeneration

**DOI:** 10.1186/1476-5926-5-2

**Published:** 2006-07-05

**Authors:** Volker Meier, Kyrylo Tron, Danko Batusic, Abderrahim Elmaouhoub, Giuliano Ramadori

**Affiliations:** 1 University of Goettingen, Department of Internal Medicine, Section of Gastroenterology and Endocrinology, Goettingen, Germany

## Abstract

**Background:**

Alpha-fetoprotein (AFP) expression can resume in the adult liver under pathophysiological conditions. Orphan nuclear receptors were supposed to regulate AFP gene expression, *in vitro*. We were interested to study the expression of AFP and orphan nuclear receptors, *in vivo*.

**Results:**

The expression of AFP gene and orphan nuclear receptors in the liver was examined in different rat models: (a) fetal liver (b) liver regeneration [partial hepatectomy (PH) with and without 2-acetyl-aminofluren treatment (2-AAF)], (c) acute liver damage [treatment with CCl_4_] and (d) acute phase reaction [treatment with turpentine oil]. After PH of 2-AAF treated rats, clusters of AFP positive cells occurred in the periportal region. In the Northern blot analysis, a positive hybridization signal for the full-length AFP-RNA was observed only in liver samples from 2-AAF treated rats after PH. In real-time PCR analysis, the full-length AFP-RNA was highly up regulated in the fetal liver (maximum at day 14: 21,500 fold); after PH of 2-AAF treated rats, the full-length AFP-RNA was also up regulated up to 400 fold (day 7 after PH). The orphan nuclear receptors were down regulated at nearly each time points in all models, also at time point of up regulation of the AFP gene.

**Conclusion:**

Expression of "fetal" AFP could be demonstrated during liver development and during proliferation of the so-called oval cells. Changes of expression of orphan nuclear receptors, however, did not correlate with AFP expression. Other regulatory pathways were possibly involved in controlling AFP expression, *in vivo*.

## Background

During severe and chronic liver damage, a subpopulation of liver cells termed oval cells was induced to proliferate. The oval cells are not typical hepatocytes; they are indeed less mature cells that can function as progenitors for either hepatocytic or ductal cell lineages. This kind of cells express alpha-fetoprotein (AFP) transcripts [1-3]. AFP is an oncofetal gene, which occurs at high rate in the yolk sac, fetal liver and intestine; but is otherwise shut off in the first weeks after birth [4,5]. In the adult liver, AFP is expressed in only very small amounts; nonetheless, AFP expression can resume in certain pathophysiological situations, such as liver regeneration (*e.g*., after surgical resection) and liver carcinogenesis (*e.g*., hepatocellular carcinoma). Increased AFP gene expression occurs, for example, in humans suffering from chronic liver disease [6-9] and was considered to be a marker for hepatocellular carcinoma [8,10]. For studying the expression of AFP, *in vivo*, different animal models of liver damage, regeneration and carcinogenesis are available. In the rat liver multiple AFP-RNA transcripts can be generated. The different AFP-RNA transcripts are differentially regulated during development, the full-length AFP-RNA [major form; 2.1 kilobases (kb)] is highly expressed in the fetal liver and the three smaller variants (1.7, 1.4 and 1.0 kb) are expressed in adult rat liver [11]. The full-length AFP-RNA, however, is strongly increased in rat livers with proliferation of a putative progenitor cell compartment [11,12]. The smaller transcript sizes of the AFP-RNA are expressed in adult rat liver and their steady state level does not change significantly in regenerating livers after partial hepatectomy (PH) or after toxic liver injury.

For understanding the mechanism of liver regeneration and hepatocarcinogenesis, it might be important some further knowledge about the regulation of the AFP gene. The transcription of the AFP gene is under the control of, at least, three enhancers and one silencing element in rat and mouse [13-15]. These factors work in a highly tissue-specific manner in the three organs derived from the endodermal layer – namely, the yolk sac, liver and intestine. In a carefully performed study, *in vitro*, and published recently, Bois-Joyeux *et al *. suggested that amounts and/or activities of the orphan nuclear receptors could modulate AFP gene expression in different pathophysiological conditions, such as liver regeneration and liver carcinogenesis [16]. Two closely related groups of transcription factors seemed to be involved in the regulation of AFP gene expression, explicitly the retinoic acid receptor-related orphan receptor (ROR) and Rev-Erb group. The first group contains three genes: ROR-α, ROR-β and ROR-γ ; the second group includes Rev-Erb A and Rev-Erb B. The ROR-α, Rev-Erb A and Rev-Erb B gene products are co-expressed in several tissues, including the heart, brain, liver and skeletal muscle [17-20]. The RORs act mostly as activators, whereas the Rev-Erb gene products most often act as transcription repressors [18,21]. Both families of transcription factors act together with cofactors, such as GRIP-1 for RORs [22] and N-CoR for Rev-Erbs [23]. As both nuclear factors bind to regulatory sites with very similar DNA sequences, there is a possibility of a cross-talk between different signalling pathways. Furthermore, both ROR-α and Rev-Erb A modulate the effect of other nuclear factors such as T3 receptor on transcription level [24]. *In vitro *experiments suggested that the AFP gene is a target of ROR-α, Rev-Erb A and Rev-Erb B. Over-expression of ROR-α stimulated the activity of the AFP enhancers in a dose-dependent manner whereas overproduction of Rev-Erb A and Rev-Erb B had a down regulating effect [16].

The aim of this study was to analyse the expression of full-length AFP-RNA, as a possible oval cell marker, and the expression of the orphan nuclear receptor (Rev-Erb A, Rev-Erb B and N-CoR) during liver regeneration [PH with or without 2-acetyl-aminofluren (2-AAF) treatment], acute liver injury [CCl_4 _treatment] and in the fetal rat liver. Therefore, we studied the distribution of the AFP-RNA (full-length as well as smaller sizes of AFP-RNA) by real-time PCR, by Northern blot and Western blot analyses, in rat liver after CCl_4 _treatment, after PH with and without 2-AAF treatment and in the fetal liver. Additionally, we studied AFP gene expression in the liver of rats after intramuscular injection of turpentine oil (model of acute phase response), as a control. At the same time points the expression of Rev-Erb A, Rev-Erb B and N-CoR were studied on mRNA level by real-time PCR.

We found that orphan nuclear receptor genes were not only down regulated when the "fetal" AFP gene was up regulated but also in cases where it was unchanged, questioning the regulatory role of those genes on expression of fetal AFP, *in vivo*.

## Results

### Expression of AFP protein in the different rat models

**Figure 1:**
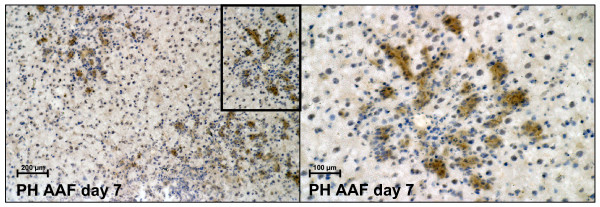
**Immunohistochemical detection of AFP in progenitor cells**. Immunohistochemical detection of AFP in progenitor cells (oval cells) in the rat liver. The right panel represents a higher magnification of the portal field admitted by black box.

Liver cryostat sections were performed from all used animal models (normal liver regeneration, liver regeneration via oval cells, acute liver injury by CCl_4_-treatment and acute phase response by turpentine oil treatment). Immunostaining with an antibody against AFP showed that AFP-positive cells could be seen only in the liver of 2-AAF treated rats after PH. AFP-positive cells were localized in clusters of oval cells, in the periportal region, at day 7 after PH with 2-AAF treatment (Figure 1). No AFP-positive cells could be seen during normal liver regeneration, after acute liver injury or after injection of turpentine oil.

### Expression of different AFP transcripts

**Figure 2:**
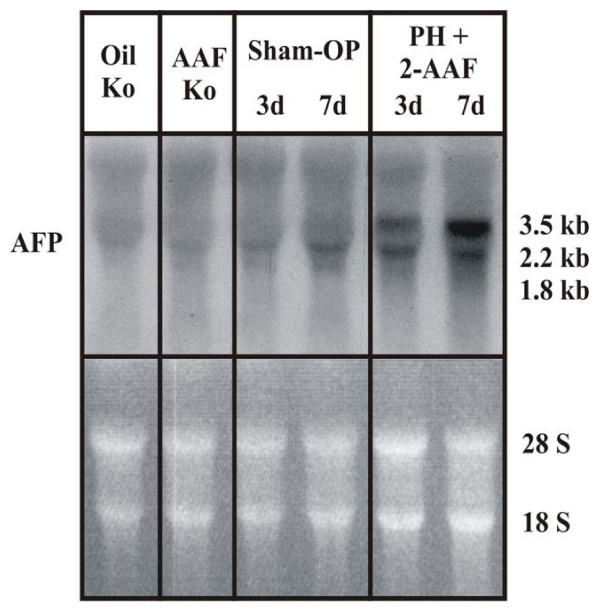
**Detection of the oval cell typical AFP-RNA product by Northern blot analysis**. Northern blot analysis was performed with liver RNA samples of 2-AAF treated rats after partial hepatectomy or after sham operation. The amplification product of the AFP1 primer pair was used as _32_P labelled probe. The first two lanes represent control samples of normal liver (Co) and 2-AAF fed animals (2-AAF-Co). In the middle RNA samples of sham operations at day 3 and day 7 are shown. The two lanes on the right site show RNA samples from day 3 and day 7 after partial hepatectomy of 2-AAF treated rats. In these samples, the oval cell typical product of 2.1 kb is detectable.

In the past, it was published by different groups that during liver regeneration by proliferation of putative progenitor cells (the so-called oval cells) the full-length AFP-RNA is highly expressed. In rats treated with 2-AAF after PH, the proliferation of the hepatocytes is blocked and the regeneration of the liver takes place by differentiation of the oval cells into hepatocytes. By Northern blot analysis (Figure 2), we could be shown that the full-length APF-RNA is expressed in this situation. For real-time PCR analysis two different primers pairs (AFP1 and AFP2) (Table 1) were designed. Real-time PCRs were performed at the same time points with those primers, interestingly in the case of primer AFP1 a strong increase of specific transcripts could be observed in comparison to the control and in case of primer AFP2 only a weak increase could be observed. To make sure that primer AFP1 can detect possibly the oval cell specific full-length AFP-RNA during liver regeneration in 2-AAF treated rats after PH, the amplification product of primer AFP1 was used as a probe for Northern blot analysis. The latter was performed with liver RNA samples of 2-AAF treated rats after PH and after sham operation. A positive hybridization signal, of about 2.1 kb, was observed only in liver samples from 2-AAF treated rats after PH; but not after sham operation (Figure 2). In order to support our assumption, the two primer pairs were also tested in fetal rat liver – here, the full-length AFP-RNA is highly expressed. Real-time PCR analysis showed a strong increased expression of full-length AFP-RNA (AFP1) with a maximum at day 14 (21,500 fold) *post coitum *and only a weak expression (582 fold) of the smaller AFP-RNA variants (AFP2) at the same time points (Figure 6) could be demonstrated. Therefore, we concluded that primer AFP1 detects the full-length AFP-RNA and the primer AFP2 detects the truncated AFP.

**Table 1:** Primer sequences and other PCR analysis related details.

**Gene**	**Primer**	**Tm (°C)**	**Number of cycles**	**Product size (bp)**
AFP1	5'-GCCCAGCATACGAAGAAAACA-3' 5'-TCTCTTTGTCTGGAAGCATTCCT-3'	60	40	179
				
AFP2	5'-ACTACTTACAAAATCTGTTCCTCATTGG-3' 5'-ATGTAAATGTCGGCCAGTCC-3'	60	40	169
				
Rev-Erb A	5'-CCATGTTTGACTTCAGCGAGAA-3' 5'-AAGTACCACTGCCGTGAAAAGG-3'	60	40	80
				
Rev-Erb B	5'-GAGCATGCCACCCCATAGAG-3' 5'-TATTGCTCCACATTCCTGGGA-3'	60	40	51
				
N-CoR	5'-TCTGCTTTGTCGTCCACACC-3' 5'-GCTGTAGCGACTTGACGGTTTA-3'	60	40	51
				
β-actin	5'-GAAATCGTGCGTGACATTAAAGAG-3' 5'-GCGGCAGTGGCCATCTC-3'	60	40	74

**Figure 3:**
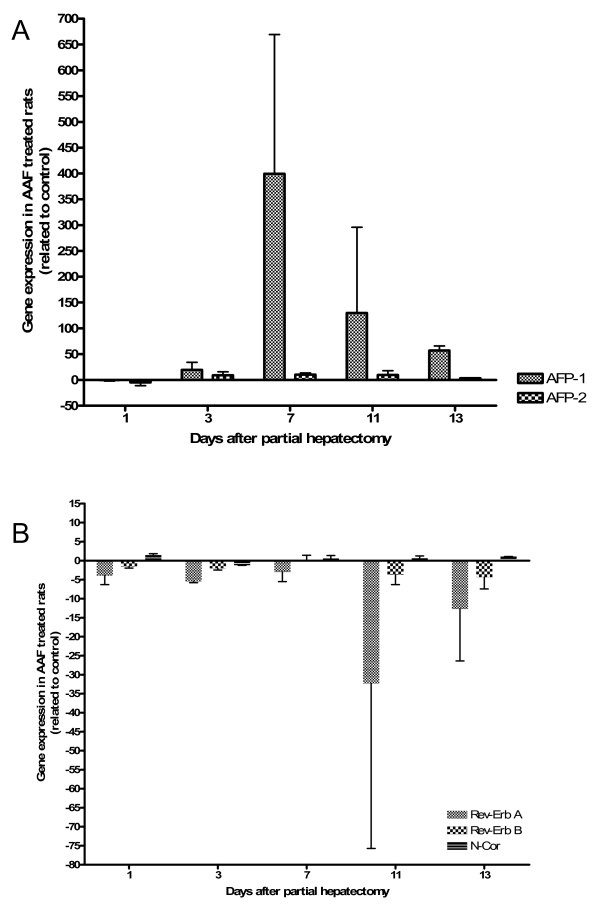
**Expression of AFP gene and Rev-Erb A, Rev-Erb B and N-CoR in liver regeneration with 2-AAF treatment**. After partial hepatectomy in 2-AAF treated rats, the expression of the AFP gene (A) and of Rev-Erb A, Rev-Erb B and N-CoR (B) were measured by real-time PCR. The mean values and the standard deviations of two different series are demonstrated. At day 7, the full-length AFP-RNA (AFP1) is maximally increased. The smaller variants of AFP-RNA (AFP2) showed no significant changes. Four days after the maximum increase of the full-length AFP-RNA Rev-Erb A is maximally decreased. Rev-Erb B is slightly decreased at each time point. N-CoR shows only slight changes in this model.

**Figure 4:**
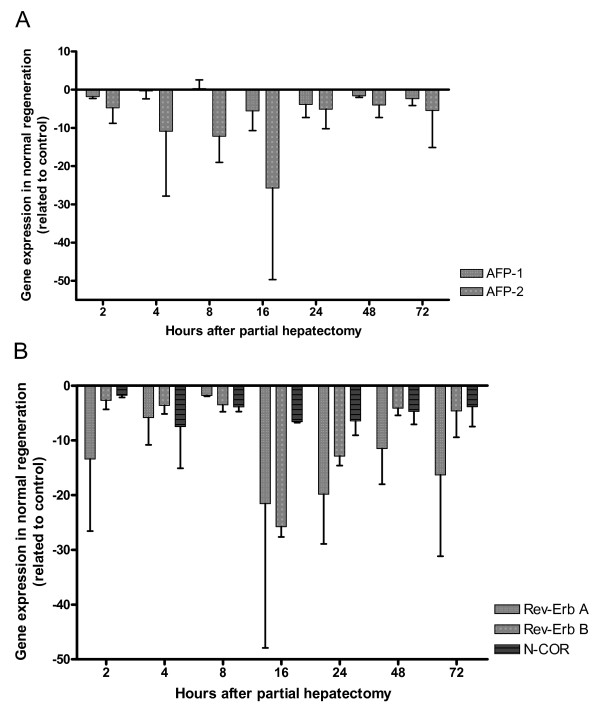
**Expression of AFP gene and Rev-Erb A, Rev-Erb B and N-CoR in liver regeneration without 2-AAF treatment**. After partial hepatectomy without 2-AAF treatment the expression of AFP gene (A) and of Rev-Erb A, Rev-Erb B and N-CoR (B) were measured by real-time PCR, in the rat liver. The mean values and the standard deviations of two different series are demonstrated. From 2 up to 16 hours, a down regulation of the smaller variants of AFP-RNA (AFP2) could be seen. Compared to 16 hours value an increase of the smaller splicing products up to -5.1 fold could be observed after 24 hours; afterwards, the level do not change up to 72 hours. The full-length AFP-RNA shows only slight changes. The genes of orphan nuclear receptors are down regulated at each time point. Rev-Erb A and Rev-Erb B are maximally down regulated after 16 hours similarly to the smaller variants of AFP-RNA (AFP2).

**Figure 5:**
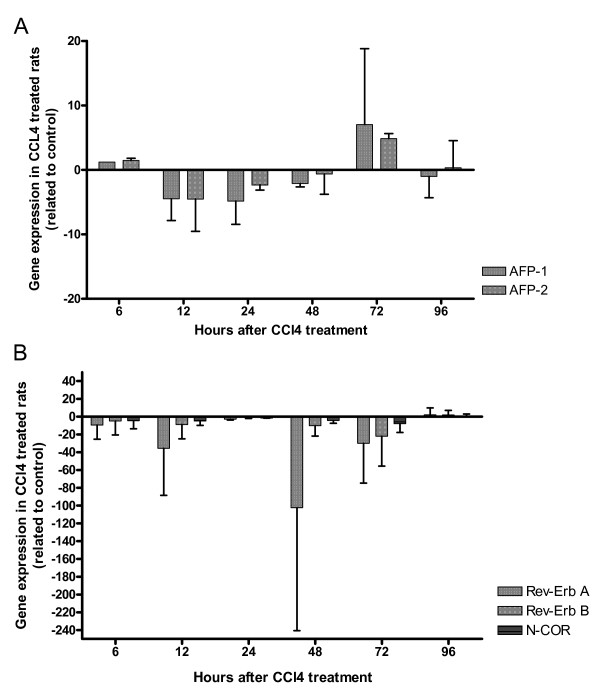
**Expression of AFP gene and Rev-Erb A, Rev-Erb B and N-CoR in the rat model of acute liver damage**. After CCl_4 _treatment, the expression of the AFP gene (A) and of Rev-Erb A, Rev-Erb B and N-CoR (B) were measured by real-time PCR. The mean values and the standard deviations of two different series are shown. A decrease of full-length AFP-RNA (AFP1; – 4.5 fold) and the smaller splicing products (AFP2; -4.5 fold) could be seen after 12 hours; afterwards, a continuous increase could be observed of both products with a maximum after 72 hours (AFP1 up to 7.0 fold and AFP2 up to 4.8 fold). A continuous down regulation of Rev-Erb A with the maximum at 48 hours could be observed, 24 hour later both AFP products were maximally up regulated. Rev-Erb and N-CoR show only slight changes in this model.

**Figure 6:**
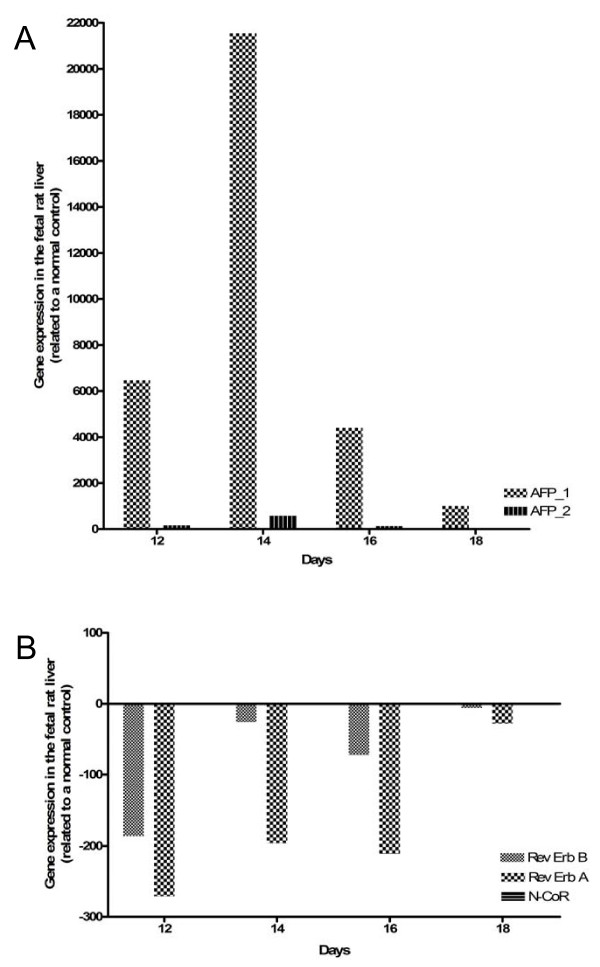
**Expression of AFP gene and Rev-Erb A, Rev-Erb B and N-CoR in fetal rat liver**. The expression of the AFP gene (A) and of Rev-Erb A, Rev-Erb B and N-CoR (B) were measured by real-time PCR in the fetal rat liver. The full-length AFP-RNA reach the maximum at day 14 (21,500 fold); afterwards, a continuous decrease up to day 18 could be observed. Rev-Erb A and Rev-Erb B are down regulated at each time point, the maximum down regulation could be seen after 12 days. N-CoR was slightly down regulated at each time point (about 1.5 fold).

**Figure 7:**
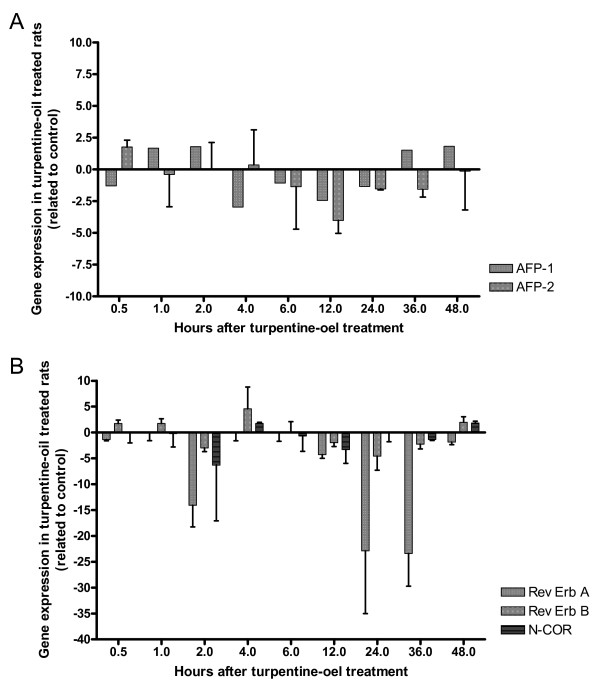
**Expression of AFP gene and Rev-Erb A, Rev-Erb B and N-CoR during acute phase reaction**. After turpentine oil treatment, the expression of AFP gene (A) and of Rev-Erb A, Rev-Erb B and N-CoR (B) were measured by real-time PCR in the rat liver. The mean values of the two different series are demonstrated in this Figure. In this model, only a slight up regulation of the AFP gene expression could be observed at 48 h. The strongest down regulation of Rev-Erb A occurs at 2, 24 and 36 hours. Compared to Rev-Erb A, Rev-Erb B gene expression was not significantly altered, *e.g*., decreased maximally by 4.5 fold at 24 hours.

Real-time PCR analysis was performed with primer AFP1, which detects the full-length AFP-RNA, and primer AFP2, which detects the smaller splicing products. The expression of the AFP gene was examined in the liver of different rat models: a) model of liver regeneration with proliferation of oval cells (PH in 2-AAF treated rats – sham operation in 2-AAF treated rats were used as appropriate controls); b) model of normal liver regeneration (PH without 2-AAF treatment – sham operation were used as appropriate controls); c) model of acute liver injury (CCl_4 _treatment of rats); d) in a model of acute phase reaction (turpentine oil treatment of rats); and e) fetal liver (Figures 3 to 7). Although the model of acute phase reaction is not a model for liver regeneration, but because dramatic changes of liver gene expression were observable, it was used as a control. We performed two different series for each model, and the mean values and the standard deviation for the AFP gene expression were shown in the figures.

In 2-AAF treated rats, the expression of the full-length AFP-RNA (AFP1) increased from day 1 (-0.2 fold) after PH to the maximum at day 7 (399 fold) after PH. From day 7 to day 13, a decrease to 56.9 fold could be observed (Figure 3). The smaller splicing products of AFP (AFP2), which are not specific for oval cells, were only slightly elevated between day 3 and day 13 after PH (Figure 3). No increase was observed in AAF-treated rats after sham operation (data not shown).

In rats undergoing PH (Figure 4) or in sham operation without AAF-treatment (data not shown) no increase of the full-length AFP-RNA (AFP1) was observed, the full-length AFP-RNA is slightly down regulated at each time point in comparison to the control. The smaller splicing products (AFP2) showed a continuous down regulation up to 16 hours with a maximum of -25.7 fold. Relating to 16 hours an increase of the smaller splicing products up to -5.1 fold could be observed after 24 hours. Afterwards, the level did not change up to 72 hours.

In rats treated with CCl_4 _a decrease of the full-length AFP-RNA (AFP1; – 4.5 fold) and the smaller splicing products (AFP2; -4.5 fold) could be seen after 12 hours. Then, a continuous increase of both products with a maximum after 72 hours (AFP1 up to 7.0 fold and AFP2 up to 4.8 fold) (Figure 5) could be observed.

In the fetal rat liver, in comparison to a normal rat liver control, a strong increase of the full-length AFP-RNA (AFP1) could be detected with a maximum at day 14 (21,500 fold); subsequently, a decrease down to 1000 fold could be seen at day 18 (Figure 6). In the case of the smaller variants of AFP-RNA (AFP2), a clear smaller increase could be observed with also a maximum at day 14 (582 fold) (Figure 6).

In the control model of acute phase reaction, only slightly changes of the full-length AFP-RNA (AFP1) and of the smaller sizes of AFP-RNA (AFP2) could be observed (Figure 7).

### Expression of Rev-Erb A, Rev-Erb B and N-CoR transcripts

The expression of the orphan nuclear receptors genes was analysed by real-time PCR in rat models of liver regeneration, acute liver injury, in the fetal liver and as control in acute phase response. For each model, we performed two different series and the mean values and standard deviations were shown in the Figures 3 to 7

After PH in 2-AAF treated rats, the full-length AFP-RNA (AFP1) was strongly increased at day 7 (399 fold) and at day 11 (129.8 fold). The smaller sizes of AFP-RNA (AFP2) were only increased at these time points to a small extent. After PH a down regulation of Rev-Erb A occurs at day 1 (-3.9 fold), day 3 (-5.5 fold), day 7 (-3.0 fold) and day 13 (-12.7). The strongest up regulation of the full-length AFP-RNA could be observed at day 7; four days later, Rev-Erb A showed the strongest down regulation (-32.3 fold). Rev-Erb B was slightly down regulated at each time point; the strongest down regulation could be observed at day 13 (- 4.3 fold). N-CoR showed only slight changes in this model (Figure 3).

After PH without 2-AAF treatment, a down regulation of Rev-Erb A, Rev-Erb B and N-CoR could be detected at each time point. The strongest down regulation of Rev-Erb A and Rev-Erb B could be observed after 16 hours (Rev-Erb A -21.5 fold and Rev-Erb B -25.7 fold). At the same time point, also the strongest down regulation of both AFP-RNA products (AFP1 and AFP2) could be detected (Figure 4).

In the case of acute liver damage after CCl_4 _treatment, a continuous decrease of Rev-Erb A from 6 hours (-9.4 fold) to the maximum at 48 hours (-102.3 fold) could be observed; but after 24 hours in relation to the control no changes could be detected. From 48 hours (-102.3 fold) to 96 hours (+2.1 fold) an up regulation of Rev-Erb A could be seen. Rev-Erb B shows continuous down regulation from 6 hours (-4.8 fold) to 72 hours (-22.1 fold), but after 24 hours in relation to the control no changes could be detected. From 72 hours (-22.1 fold) to 96 hours (+2.0 fold) an up regulation of Rev-Erb B could be seen. N-CoR shows only slight changes in this model (Figure 5).

In the fetal liver Rev-Erb A and Rev-Erb B are down regulated at each time point, the maximum down regulation was reached at day 12 (Rev-Erb A: 270 fold/Rev-Erb B: 186 fold), two days before the maximum up regulation of full-length AFP RNA. N-CoR was slightly down regulated at each time point (about 1.5 fold) (Figure 6).

In the rat model of acute phase reaction, the strongest down regulation of Rev-Erb A occurs at 2 hours (-14.1 fold), 24 hours (-22.8 fold) and 36 hours (-23.4 fold) after turpentine oil treatment. A continuous increase of Rev-Erb B could be seen from 30 minutes (+1.7 fold) to 4 hours (+4.7 fold) after treatment with turpentine oil, with exception of 2 hours; at this time point, Rev-Erb B was down regulated. From 4 hours (+ 4.7 fold) to 24 hours (- 4.6 fold) a continuous down regulation of Rev-Erb B could be detected; after that, Rev-Erb B was up regulated again (48 hours: + 2.0 fold) (Figure 7).

### Western blot analysis of Rev-Erb A and Rev-Erb B expression in the different rat models and immunhistological staining

The results of Rev-Erb A and Rev-Erb B expression should be verified on protein level. Therefore, Western blot analyses were performed with samples of PH rats with and without 2-AAF treatment. Actually, only human specific antibodies against Rev-Erb A and Rev-Erb B are available, whereas Rev-Erb B should cross-react with the rat. Those available antibodies have been not tested from the manufactures in "natural samples", they have only been tested in CHO-cells transfected with full-length Rev-Erb A and Rev-Erb B transcript. The protein size of the Rev-Erb A and Rev-Erb B is 70 kDa. In the Western blot analysis, no specific band could be detected at 70 kDa (data not shown). There are two possible reasons for our finding: (1) the antibodies are not specific for rat samples or (2) the protein level is too low, because Rev-Erb A and Rev-Erb B are down regulated in our models. Samples for Western blot analysis were also extracted from rat hepatoblasts and human hepatoma cells (Hep G2, Hep 3B); but also in these cell-lines no specific band could be detected at 70 kDa.

The same antibodies were also used for immunohistological techniques. Different immunohistological techniques (standard indirect peroxidase staining and enhanced peroxidase-based detection (Envision; DAKO, Glostrup, Denmark)) were applied on differently fixated liver sections (methanol-acetone fixated and paraformaldehyde fixated) proved unsuccessful in the rat (data not shown).

### Western blot analysis of AFP expression in the different models

**Figure 8:**
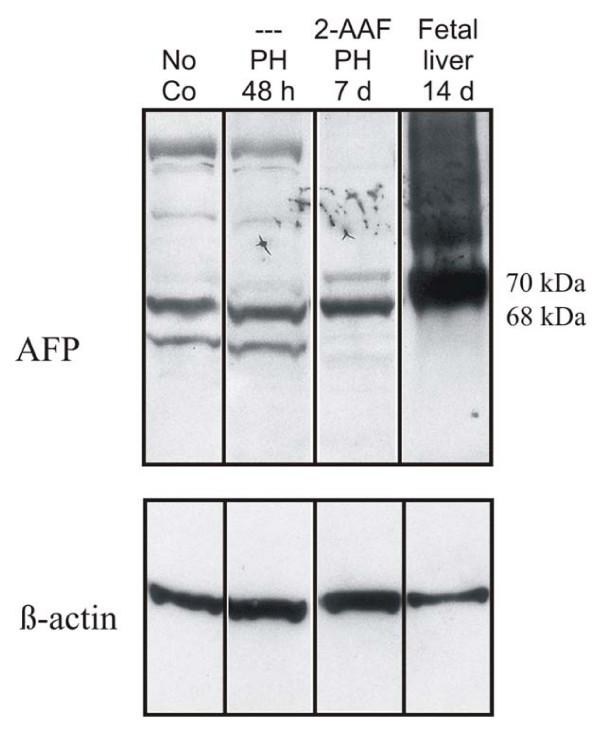
**Western blot analysis of AFP expression in the rat liver**. The fetal AFP-RNA is translated into a 68 kDa and 70 kDa protein. The smaller AFP-RNA transcripts are translated into smaller protein products (58 kDa, 54 kDa and 44 kDa). The strongest expression of fetal AFP protein could be detected in the fetal liver. After PH and 2-AAF treatment, a clearly weaker expression of proteins with a size of 68 kDa and 70 kDa could be seen; but no smaller protein products could be observed. In the case of the normal PH and of the normal control (no co), beside a weak 68 kDa signal also a smaller protein product could be detected.

The expression of the AFP protein was analysed by Western blot technique. The full-length AFP-RNA, which is highly expressed in the fetal liver, is translated into 68 kDa and 70 kDa proteins, whereas the smaller AFP-RNA variants, which are expressed in the adult liver, are translated into smaller proteins of 58 kDa, 54 kDa and 44 kDa [11]. Firstly, we performed Western blot analysis with fetal liver (days 12, 14, 16 and 18) for verifying whether the used AFP antibody detected the full-length AFP-RNA protein of 68 kDa and 70 kDa. In the Western blot analysis at day 14, a strong band could be detected at 68 kDa and 70 kDa (Figure 8).

Then, we performed Western blot analysis with samples of rat livers after PH with and without 2-AAF treatment. In the samples of rats treated with 2-AAF, only a weak band could be detected at a protein size of 68 kDa and 70 kDa, in comparison to the fetal liver with no changes at the different time points.

## Discussion

AFP is a fetal glycoprotein produced by the yolk sac, fetal liver and intestine, but its synthesis is shut off after birth [25]. AFP is used as a serum marker for diagnosis of hepatocellular carcinoma [8,26]. Elevated serum AFP level has also been observed in various chronic liver diseases, where continuous hepatocellular damage occurs and hepatocellular regeneration is supposed to take place [27]. In this case, mature hepatocytes are supposed to differentiate back to hepatoblasts and to be responsible for the increased serum levels of fetal AFP. Nevertheless, and in one study, AFP expression was not found in regenerating human liver after PH [28]. Although many investigations on the function of AFP had been carried out, the regulation and biological role of AFP is still unclear.

The adult liver can regenerate by mature hepatocytes reentering into cell cycle after surgical resection or injury. However, this proliferative response is often impaired in chronic liver disease. Activation of a facultative stem cell compartment (oval cells) is considered as an alternative mechanism for regeneration in chronic liver disease [29-31]. In rodent models of hepatocarcinogenesis and non-carcinogenic injury models, it has been suggested that oval cells might represent a facultative hepatic progenitor/stem cell compartment proliferating and differentiating both into hepatocytes and biliary epithelial cells, under certain conditions [32-34]. PH combined with 2-AAF feeding is a traditional model to activate oval cells, in rat liver [35,36]. Several markers for oval cells have been suggested. However, many of those markers are not specific for oval cells; but are expressed by biliary epithelial cells (CK-7, CK-19) and by hematopoietic cells (c-kit, Thy-1) [37,38]. AFP gene expression seems to represent the most reliable marker for oval cells, because it is widely studied in human and animal models and it is not expressed by other cell types, with the exception of some hepatocellular carcimoma cells [11]. We studied the expression of the full-length AFP-RNA (major form, 2.1 kb mRNA) and of the smaller splicing variants during liver regeneration, acute liver injury, in the fetal liver and under conditions of acute phase response in the rat. Northern blot analysis showed that the full-length AFP-RNA occurs only after PH in 2-AAF treated rats; but not after sham operation of 2-AAF treated rats or in normal controls.

Immunohistochemistry revealed clusters of AFP-positive cells in the periportal region of 2-AAF treated rats after PH at day 7, the typical area in which early proliferation of oval cells takes place [39]. At the same time points, real-time PCR analysis were performed with the two different AFP primers (AFP1 and AFP2). Interestingly, and in the case of primer AFP1, a strong increase could be observed in comparison to the normal control and in case of primer AFP2 only a weak increase could be observed. For verifying whether primer AFP1 could possibly detect the full-length AFP-RNA during liver regeneration in 2-AAF treated rats after PH, the amplification product of primer AFP1 was used as a probe for Northern blot analysis. This was performed with liver RNA samples of 2-AAF treated rats, after PH or after sham operation. A positive hybridization signal of about 2.1 kb was observed only in liver samples from 2-AAF treated rats after PH; but not after sham operation (Figure 2). In order to support our assumption, the two primer pairs were also tested in fetal rat liver. In the fetal liver the full-length AFP-RNA is highly expressed. In real-time PCR analysis, a strongly increased expression of full-length AFP-RNA (AFP1) with a maximum at day 14 (21,500 fold) could be shown. Only a weak expression (582 fold) of the smaller AFP-RNA variants (AFP2) at the same time points was seen (Figure 6). Fetal AFP was not detected in the liver of animals after normal PH. In this vein, we concluded that primer AFP1 detects the full-length AFP-RNA and the primer AFP2 detects the truncated AFP. The full-length AFP-RNA is translated into a 68 kDa and 70 kDa protein. The AFP antibody we used for the Western blot analysis detected the 68 kDa and 70 kDa protein in fetal liver; a strong signal could be seen at those sizes. But in case of rats treated with 2-AAF and PH, only weak signals could be detected by 68 kDa and 70 kDa at the different time points; furthermore, smaller protein products could be not seen, however, no strong changes could be observed on protein level. The possible explanation for these findings is that the amount of the full-length AFP-RNA protein is very low in comparison with that contained in the fetal liver, therefore, no changes could be detected in those samples. The difference between fetal AFP gene expression in the fetal liver (maximum at day 14: 21,500 fold) and in the liver of 2-AAF treated animals after PH (maximum at day 7: 400 fold) was about 54 fold.

In the CCl_4 _rat model of acute liver damage an increase of full-length AFP-RNA (AFP1) up to 7 fold, and of the smaller splicing variants (AFP2) up to 5 fold could be observed after 72 h hours. Our results are similar to those of Lemire *et al *. [11]; in fact, they described also the maximum increase of the full-length AFP-RNA after 72 hours and of the smaller transcripts up to 4 fold at the same time point.

In the model of normal liver regeneration (PH without 2-AAF treatment), the full-lenght AFP-RNA transcript (AFP1) is slightly down regulated at each time point, no really strong changes could be detected. But in case of the smaller AFP-RNA transcripts (AFP2), a continuous down regulation from 2 hours (- 4.7 fold) up to 16 hours (-25.7 fold) could be seen. Compared to 16 hours, an increase of the smaller splicing products up to -5.1 fold could be observed after 24 hours; then, the level of do not change up to 72 hours. The slight changes in the case of full-length AFP-RNA and the stronger chances in case of the smaller variants of AFP-RNA, as described above, support the theory that, in the adult liver, normally only the smaller variants were expressed and no regeneration takes place via oval cells. No induction of the full-length AFP-RNA or the smaller AFP-RNA transcript could be observed after induction of an acute phase reaction by turpentine oil in our control model.

So far, the exact regulation of AFP is still unknown; therefore, another aim of our study was to analyse the expression of the orphan nuclear receptors genes (Rev-Erb A, Rev-Erb B and N-CoR) suggested to control the expression of AFP negatively. Transfection experiments performed on human Hep G2 hepatoma cells showed that overproduction of ROR-α stimulated the activity of AFP enhancers in a dose-dependent manner, whereas that of Rev-Erb A and Rev-Erb B had the opposite effect. It was suggested that the activities of those orphan receptors in cells of hepatic or endodermal origin could modulate AFP gene expression, in response to a variety of developmental or carcinogenic stimuli [16]. In our study, the expression of the orphan receptors Rev-Erb A, Rev-Erb B and N-CoR was investigated, *in vivo*, and in different animal models of liver regeneration, liver injury, acute phase reaction and in the fetal liver. In these models, a down regulation of the orphan receptors (Rev-Erb A, Rev-Erb B and N-CoR) could be seen, at nearly all time points. The expression of the full-length AFP-RNA was up regulated after PH in 2-AAF treated rats (maximum at day 7: 400 fold) (Figure 3) and after acute liver damage (CCl_4 _treatment; maximum after 72 hours: 7 fold) (Figure 5) at the same time points Rev-Erb A and Rev-Erb B were always down regulated. That means that at those time points no inhibitory effect of Rev-Erb A and Rev-Erb B of the AFP gene expression takes place. However, no AFP up regulation was observed in the other models when the genes of the orphan nuclear receptors were also down regulated. But the reason why the orphan nuclear receptors were down regulated at nearly all time points still remains unclear. Possible explanations for those findings are, on the one hand, that the regulation of the AFP gene and the orphan nuclear receptors differs in different organisms and, on the other hand, that *in vivo *some other additional factors may participate in the regulation of the AFP gene expression and of the orphan nuclear receptors. Because no rat specific antibodies against Rev-Erb A and Rev-Erb B were commercially available, an attempt to verify the results of the Real-time PCR at protein level could be not performed. Therefore, and as an alternative, we tried Western blot analysis and immunohistological staining with human antibodies; but no specific signal could be detected. Possible explanations for these findings are: (1) the antibodies do not react with the rat samples or (2) the protein level is too low. For addressing to the second point, we studied the relationship between full-length (fetal AFP) and adult (smaller variants) AFP on RNA level and, then, on protein level. The difference between expression of the full-length AFP-RNA in the fetal rat liver (maximum at day 14: 21,500 fold) and after PH in 2-AAF treated rats (maximum at day 7: 400 fold) was about 54 fold. On protein level, studied by Western blot analysis, we could reproduce these results. In comparison to the fetal liver, with a strong specific signal, the full-length AFP protein was only barely detectable in the total liver proteins of the rat livers after PH with after 2-AAF treatment. Therefore, it is understandable that the detection of transcription factors like the orphan nuclear receptors became even more difficult, because they are normally low expressed and in this study additional still down regulated.

## Conclusion

The proliferation of oval cells could be demonstrated in 2-AAF treated rats after PH, on RNA level by real-time PCR with a specific primer pair (AFP1) and on protein level by immunostaining. Only traces of the oval cell specific AFP transcripts could be observed in regenerating livers without 2-AAF treatment. The orphan receptor genes, which could influence the AFP gene expression *in vitro*, showed in the oval cell model (PH with 2-AAF treatment) and, in the model of acute liver damage, a down regulation of Rev Erb A and Rev Erb B at the same time points of the up regulation of the AFP gene expression. Yet, down regulation of the orphan nuclear receptor genes could also be observed nearly at all time points in the different model; therefore, the correlation of AFP gene and orphan nuclear receptors is not really clear. Further investigations will be necessary for understanding the regulation of AFP gene in rodent models of oval cell proliferation and in human hepatocellular carcinomas. Eventually, novel regulatory factors of AFP gene expression could be utilized as early markers of hepatocarcinogenesis.

## Materials and methods

### Animals

Male Wistar rats (180–200 g) were provided by Winkelmann (Borchen, Germany) and used for animal models of acute liver injury or acute phase response and further used for studying liver regeneration after PH. Two series were performed for each model. The animals were kept with food and water, *ad libitum*. For each model of liver regeneration and liver injury, two different series were performed. The animals were kept according to our institution's and the National Institutes of Health guidelines.

### Different rat models of liver regeneration

Proliferation of hepatic progenitor cells (oval cells) was studied in rats treated with 2-AAF, after PH. Rats received 1.5 mg 2-AAF daily by intragastric gavage and PH or sham operation was perfomed on the sixth day. On the day of surgery and on the first postoperative day, 2-AAF was not given. PH was performed under ether anaesthesia by midventral laparotomy with 70% liver resection. A control animal was subjected to sham operation by the same operator. Under similar conditions, sham operations consisted of a midventral laparotomy, gentle manipulation of the liver and surgical closure of the abdomen. Treatment with 2-AAF was restarted on the second postoperative day and continued for another 5 days to a total dose of 15 mg per rat. Rats were sacrificed on the day of surgery (control), day 1 after PH or sham, and days 3, 7, 11 and 13 (each PH and sham operation). Normal liver regeneration without 2-AAF treatment was studied after standard PH or sham operation. Rats were sacrificed on the day of surgery (control), and 2, 4, 8, 16, 24, 48 and 72 hours, after PH or sham operation. Livers were snap-frozen in liquid nitrogen and stored at -80°C.

### Rat model of acute liver damage

Acute liver damage was induced in 8 weeks old Wistar rats by oral administration of a CCl_4_/maize oil solution (50% v/v) according to Yokoi [40], as previously described [41]. For studying the time kinetics, rats were sacrificed 6, 12, 24, 48, 72 and 96 hours, after a single CCl_4 _administration. Livers were snap-frozen in liquid nitrogen and stored at -80°C.

### Rat model of acute phase reaction

Acute phase reaction was induced in male Wistar rats by injection of 2.5 ml/kg turpentine oil into the right and left hind limp, intramusculary, as reported [42]; control animals received no injections. Liver tissue was removed after 30 minutes, and 1, 2, 4, 6, 12, 24, 36 and 48 hours, after injection. Livers were snap-frozen in liquid nitrogen and stored at -80°C.

### Immunohistology

Rat liver cryostat sections were fixed in methanol/acetone, at -20°C. After inactivation of endogeneous peroxidases with 0.01 M glucose, 250 U glucose oxidase and 1 mM sodium azide in phosphate-buffered saline (PBS), sections were incubated with fetal calf serum, for 30 minutes at room temperature. The sections were incubated with a rabbit antiserum against human AFP (DAKO, Glostrup, Denmark) diluted 1:50 in PBS, at 4°C overnight. After washing in PBS, sections were incubated with a peroxidase-conjugated secondary antibody (Swine anti-Rabbit, DAKO, Glostrup, Denmark), for 60 minutes at room temperature. As a substrate for immunostaining, 3,3'-diaminobenzidine tetrahydrochloride was used.

### Western blot analysis

Lysates were prepared by homogenization of tissue at 4°C in 20 mM Tris-HCl (pH 8.0), 5 mM EDTA, 3 mM EGTA, 1 mM DTT, 1% SDS, 1 mM PMSF, and protease inhibitor cocktail (Sigma-Aldrich Inc.). Samples were cleared by centrifugation at 15,000 g for 15 min at 4°C, and the protein concentration was measured by BCA assay (Pierce, Rockford, IL, USA), using bovine serum albumin, as standard. Protein lysates were separated on SDS-polyacrylamide gels, electrotransferred to polyvinylidene difluoride membranes (Invitrogen, USA), probed with primary antibodies overnight. The appropriate peroxidase-conjugated secondary antibodies (DAKO, Glostrup, Denmark) in a dilution of 1:5,000 were then added and incubation continued for 1 hour, at room temperature. Bound antibodies were visualized using chemiluminescent substrate (ECL; Amersham-Pharmacia, UK). Equal loading was previously controlled by transient Ponceau S staining. The primary antibodies included: human anti-AFP (DAKO, Glostrup, Denmark), human anti-RevErbα/NR1D1 (R&D Systems, Wiesbaden, Germany) and human anti-RevErbβ/NR1D2 (R&D Systems, Wiesbaden, Germany). Probes were also extracted from rat hepatoblasts and hepatoma cells (Hep G2, Hep 3B).

### Isolation of total RNA

Frozen liver samples were lysed in 3 ml guanidinium isothiocyanate buffer [43], as described elsewhere [44]. Subsequently, total cellular RNA was isolated by cesium chloride density gradient centrifugation [45] and the RNA concentration was determined photometrically.

### Northern blot analysis

Total RNA was resolved by agarose gel electrophoresis, transferred on nylon membranes and hybridized with cDNA probe specific for the full-length AFP mRNA (AFP1 fragment). The cDNA probe was 32P-labed by nick translation or by random priming. Hybridiziation was performed under high-stringency conditions for 2 hours, at 68°C, using the QuickHyB^® ^(Stratagene, La Jolla, California). Posthybridization washes were performed 2 times for 15 minutes each at room temperature, and 1 time for 5 to 15 minutes at 60°C in two fold standard sodium citrate containing 0.1% sodium dodecyl sulfate (SDS). Nylon filters exposed to Kodak X-omat x-ray films, at -80°C.

### Quantification of gene expression by real-time PCR

The reverse transcriptase reaction was carried out in 0.1 M Tris chloride (pH 8.3), 10 mM magnesium chloride, 10 mM dithiothreitol using 1 mM of each deoxynucleotide (dATP, dCTP, dGTP, dTTP, Boehringer Mannheim, Germany), 200 units of Moloney murine leukaemia virus reverse transcriptase (M-MLV-RT, Invitrogene, Karlsruhe, Germany), and 16 units of RNasin in a total volume of 20 μl for 1 hour at 40°C using 1 μg denatured total RNA and Oligo (dT)_15 _primer.

The expression of mRNAs was measured by real-time PCR, a method to precisely quantify mRNA. The cDNA was amplified with a set of gene-specific primers and SYBR Green Master Mix (Applied Biosystems, Foster City, California), according to the manufacturer's protocol, in a PRISM 7000 Sequence Detection system (Applied Biosystems, Foster City, California). Primers used in the real-time PCR experiments are summarized in Table 1. Amplified PCR products were monitored by measuring the increase in fluorescence caused by the binding of SYBR Green dye to double-stranded DNA. Real-time PCR was performed twice for each sample. The amount of comparative expression level of the target, normalized to an endogenous reference (β-actin) and relative to a calibrator (control animal at time point 0 h), is given by 2^-ΔΔC ^_T_.

## Competing interests

The author(s) declare that they have no competing interests.

## Authors' contributions

VM was responsible for the primer design, carried out the Northern blot analysis and real-time PCR analysis, and drafted the manuscript. KT carried out experiments with the rat models for acute liver damage (treatment with CCl_4_) and for acute phase reaction (treatment with turpentine oil). DB carried out the experiments with rat models for liver regeneration (PH with and without 2-AAF treatment) and the immunostaining with an antibody against AFP. AE performed the experiments with the fetal rat liver. GR conceived this study, and participated in the design and coordination of the different experiments and helped to draft the manuscript.
